# Detailed association between B‐type natriuretic peptide levels and ventricular tachyarrhythmia

**DOI:** 10.1002/clc.24124

**Published:** 2023-08-31

**Authors:** Naoya Kataoka, Teruhiko Imamura

**Affiliations:** ^1^ Second Department of Internal Medicine University of Toyama Toyama Japan

To the Editor:

It is well known that advanced heart failure is associated with the development of ventricular tachyarrhythmia (VT). However, it is uncertain whether the elevated *N*‐terminal pro‐B‐type natriuretic peptide (NT‐pro BNP) level is a predictor of the incidence of VT in patients with implantable cardioverter defibrillator (ICD). Sourour and colleagues demonstrated that the elevated NT‐pro BNP levels were associated with the risk of incident VT, particularly in patients with a secondary prevention ICD indication.^1^ Several concerns have been raised.

Patients with a variety of baseline diseases were included in the authors' study.^1^ According to the SCD‐HeFT trial, the efficacy of ICD varied depending on the baseline diseases.^2^ For example, the efficacy of ICD was greater in patients with ischemic heart failure than in those with nonischemic heart failure. The long‐term efficacy of ICD in preventing all‐cause death was significant in the New York Heart Association class II cohort but nonsignificant in the New York Heart Association class III cohort. Could the authors perform a sub‐analysis to standardize the patients' baseline diseases?

The long‐term effect of ICD is another concern. In the SCD‐HeFT trial, the preventive effect of ICD gradually decreased from 6 years after the device implantation until 10 years when its preventive impact decreased as low as those of amiodarone.^2^ In the authors' study, the timing between ICD implantation and NT‐pro BNP measurement was not stated, which should have a significant prognostic impact.^1^

In the authors' study, the change in NT‐pro BNP levels was not associated with the incidence of VT.^1^ There are two major mechanisms in the development of VT: monomorphic VT and polymorphic VT.^3^ Monomorphic VT is caused by the re‐entrant circuit due to cardiac fibrosis and can develop without any triggers. On the contrary, polymorphic VT is caused by the injured Purkinje fibers and can be caused by the progression of heart failure. Sub‐analysis of these different origins of VT may further clarify the prognostic impact of NT‐pro BNP levels. The requirement of electrical defibrillation or anti‐tachycardia pacing would be useful in distinguishing these two origins.

## References

[1] Sourour N , Riveland E , Naesgaard P , et al. N‐terminal pro‐B‐type natriuretic peptide for prediction of ventricular arrhythmias: data from the SMASH study. Clin Cardiol. 2023. 10.1002/clc.24074 PMC10436794 37400982

[2] Poole JE , Olshansky B , Mark DB , et al. Long‐term outcomes of implantable cardioverter‐defibrillator therapy in the SCD‐HeFT. JACC. 2020;76(4):405‐415. 32703511 10.1016/j.jacc.2020.05.061

[3] Masuda K , Nogami A , Kuroki K , et al. Conversion to Purkinje‐related monomorphic ventricular tachycardia after ablation of ventricular fibrillation in ischemic heart disease. Circ: Arrhythm Electrophysiol. 2016;9(9):e004224. 27635070 10.1161/CIRCEP.116.004224

